# Development of a biomolecular approach to identify sperm functions and fertility using sperm RNAs

**DOI:** 10.3389/fcell.2023.1308167

**Published:** 2023-12-05

**Authors:** Won-Ki Pang, Yoo-Jin Park, Myung-Geol Pang

**Affiliations:** Department of Animal Science and Technology and BET Research Institute, Chung-Ang University, Anseong, Gyeonggi-do, Republic of Korea

**Keywords:** male infertility, pig, sperm motility, capacitation, fertilization

## Abstract

Infertility affects a significant percentage of couples worldwide, and male factors contribute significantly to this problem. Traditional assessments of male fertility rely primarily on parameters such as sperm motility, morphology, viability, and concentration. However, these metrics often do not provide a comprehensive understanding of sperm function, which is critical not only for fertilization but also for successful embryo development. Herein, we used porcine spermatozoa as a model to investigate the potential of sperm RNA markers in assessing various aspects of sperm function from motility to fertility. Using artificial insemination, we evaluated male fertility based on the litter size of sows inseminated with sperm from 20 boars. In addition, we measured parameters such as weaning rate, death births, live births, and mummy births. Sperm functional parameters, including motility and kinematics, were assessed before and after *in vitro* capacitation. Finally, correlations between various sperm functional parameters and sperm RNA markers were explored. Our results revealed interesting relationships between sperm functional parameters. While motility and kinematics were correlated, they were not correlated with sperm capacitation status. Surprisingly, no significant correlations were found between these parameters and male fertility. However, specific parameters of sperm capacitation status after *in vitro* capacitation were correlated with weaning rate and live births, highlighting their importance in predicting successful fertilization. Further analysis of sperm RNA markers identified genes related to male fertility, including IZUMO1, known for its role in sperm-egg fusion. These genes showed correlations with motility, capacitation, and fertilization parameters, shedding light on their potential roles in sperm function. In conclusion, our study demonstrates that sperm RNA markers hold promise for the diagnosis and prognosis of various aspects of sperm function, providing valuable insights into male infertility. These markers may serve as diagnostic tools to improve our understanding of male fertility problems, ultimately benefiting couples struggling with infertility.

## 1 Introduction

Fifteen percent of couples at reproductive age suffer from infertility worldwide (Dyer, 2009). Male factors solely contribute 20%–30% to infertility, which could be the cause in approximately 50% of couples (Patel et al., 2018; Agarwal et al., 2021a). The evaluation of male infertility in the biomedical field is currently based on the World Health Organization (WHO) criteria using semen (Barratt et al., 2017). The WHO criteria mostly depend on the sperm morphology, viability, motility, and concentration in semen.

The spermatozoon is a highly specialized motile cell that travels through the female reproductive tract to transfer the paternal genome into the oocyte. Sperm motility is linked to spermatozoa survival in the female reproductive tract and directs fertility. Therefore, diverse sperm assessment, including the WHO criteria, is mainly focused on motility. However, sperm function is not only conveyed to the oocyte but also has a substantial contribution in embryo formation (Ostermeier et al., 2004). Therefore, beside using the WHO criteria, not only motility but also diverse sperm parameters like DNA fragmentation, oxidative stress, and reactive oxygen species are assessed in the biomedical field (Agarwal et al., 2021b).

While sperm motility is critical in assessing male fertility, a wide range of parameters should be measured due to the presence of idiopathic male infertility where sperm morphology and motility may appear normal (Farkouh et al., 2022; Corsini et al., 2023). Recent advances in sperm motility analysis techniques allow precise analysis of sperm motility kinetics, allowing the assessment of individual sperm movements (Dai et al., 2021). In addition to sperm motility, various parameters such as sperm morphology, hyperactivated motility, movement kinematics, DNA integrity, membrane potential and capacitation status are measured to assess a man’s potential fertility (Khatun et al., 2018). However, these techniques are not widely used in the medical and industrial fields because reliance on a single parameter cannot accurately predict fertility in heterogeneous sperm populations.

Therefore, in addition to sperm functional parameters, various markers are being developed and numerous studies are underway to validate their practical use (Jodar et al., 2017; Burl et al., 2018; Agarwal et al., 2020; Hernandez-Silva et al., 2022; Yang et al., 2022). Among these, transcriptomic markers are particularly promising due to the well-developed high-throughput techniques used for their analysis and the availability of various on-site validation tools. Recent research has also evaluated the predictive ability of sperm RNA for fertility. In particular, there are studies evaluating the ability of specific genes in porcine spermatozoa to predict fertility (Pang et al., 2020; Pang et al., 2022a; Pang et al., 2022b). Large-scale data generated by high-throughput techniques provide a comprehensive view of the transcriptome landscape. However, before entering the preclinical phase in the biomedical field, it is of utmost importance to perform functional evaluations. Validating the predictive power of RNA markers derived from transcriptomic data is a critical step that should be taken before proceeding to clinical evaluation.

Pigs share similarities with humans in terms of sperm morphology, size, motility patterns, and physiological mechanisms (Gu et al., 2019). When studying the function of mammalian spermatozoa, the advantage of using pigs is their polytocous nature, which allows statistically significant analysis of parameters measured in a heterogeneous sperm pool against well-established artificial insemination (AI) outcomes.

In this study, we used porcine spermatozoa to investigate the potential of transcriptomic markers in assessing mammalian sperm function. We evaluated sperm motility, movement kinematics, and *in vitro* capacitation induction in a sample pool with well-documented fertility data and compared the results with known sperm RNA markers.

## 2 Materials and methods

### 2.1 Animal care

All procedures involving pigs were approved by the Institutional Animal Care and Use Committee of Chung-Ang University (Approval No. 2017-00018) and performed in accordance with the corresponding guidelines. All methods were performed according to relevant guidelines and regulations. Environmental conditions for the pigs were controlled, with a temperature maintained between 20°C ± 5°C, proper ventilation, and a lighting schedule of 16 h of light followed by 8 h of darkness. Appropriate feed was provided for boars and sows, and water was provided *ad libitum*.

### 2.2 Measurement of male fertility

The AI results were used as parameters to assess a boar’s fertility status. Boar semen samples were collected using the gloved hand technique and diluted with Beltsville thawing solution at a ratio of 100 mL per 30 × 10^6^ sperm after collection. The diluted samples were then stored at 17°C until used for insemination. To control for potential female-related factors, we specifically selected sows with parities between 2 and 8 for the AI procedure (Schulze et al., 2013).

### 2.3 Boar sperm preparation

After collection, semen samples were immediately placed in a container maintained at a constant temperature of 17°C until used in subsequent processing steps (Kwon et al., 2015). All semen samples were then centrifuged at 500 *g* for 20 min using a discontinuous gradient of 70% (v/v) and 35% (v/v) Percoll (Sigma-Aldrich, St. Louis, MO, United States). This centrifugation step was designed to remove seminal plasma and any non-motile or non-viable spermatozoa (Flesch et al., 1999). The isolated viable spermatozoa were then incubated for 30 min at 37°C in modified tissue culture medium 199 containing 0.91 mmol/L sodium pyruvate, 3.05 mmol/L D-glucose, 2.92 mmol/L calcium lactate, and 2.2 g/L sodium bicarbonate (Sigma-Aldrich) (Oh et al., 2010; Kwon et al., 2015). To induce *in vitro* capacitation, the modified tissue culture media were supplemented with 10 μg/mL heparin and 100 μL/mL fetal bovine serum.

### 2.4 Computer-assisted sperm analysis

Analysis of boar sperm motility (%) and movement kinematics was performed using a Computer-Assisted Sperm Analysis system (SAIS-PLUS VERSION 10.1; Medical Supply, Seoul, Korea) (Paston et al., 1994). After the incubation period, a 10 μL aliquot of the sperm sample was placed on a Makler counting chamber purchased from Sefi Medical Instruments (Sefi Medical Instruments, Haifa, Israel). This chamber was then placed on a heating block prewarmed to 37°C (Sukcharoen et al., 1994; Yoon et al., 2015). Various sperm parameters, including sperm motility, hyperactivated motility (HYP), curvilinear velocity (VCL), straight-line velocity (VSL), average path velocity (VAP), average amplitude of lateral head displacement (ALH), beat cross frequency (BCF), linearity (LIN), and wobble (WOB), were determined (Oh et al., 2010). To capture sperm motility and other motion kinetics, we used a frame rate of 60 frames per second (fps) for a duration of 0.5 s in each field containing at least 20 viable sperm for analysis. Each sample was observed using phase contrast microscopy with a ×10 objective (Farrell et al., 1998).

### 2.5 Combined hoechst 33258/chlortetracycline capacitation status fluorescence assessment

The capacitation status of boar spermatozoa was assessed using a double staining technique with Hoechst 33258 and chlortetracycline (CTC) fluorescence staining (Kwon et al., 2015; Kwon et al., 2018). First, the samples were centrifuged at 400 *g* for 10 min at room temperature. After removing the supernatant, 135 μL phosphate-buffered saline (PBS) and 15 μL H33258 solution were added to the pellets. Samples were gently mixed and incubated for 10 min at room temperature. Any excess dye was neutralized with 250 μL of 2% (w/v) polyvinylpyrrolidone (Sigma-Aldrich) in PBS. After further centrifugation at 400 *g* for 10 min, the supernatant was discarded, and the pellet was reconstituted in a solution containing 600 μL PBS and 600 μL CTC fluorescence solution (consisting of 750 mM CTC in 5 μL buffer containing 20 mM Tris, 130 mM sodium chloride, and 5 mM cysteine at pH 7.4) from Sigma-Aldrich (Kwon et al., 2013). The stained samples were then examined with a Microphot-FXA microscope (Nikon, Tokyo, Japan) under epifluorescence illumination using ultraviolet BP 340–380/LP 425 and BP 450–490/LP 515 excitation/emission filters for Hoechst 33258 and CTC, respectively. The capacitation status was quantified by observing approximately 400 spermatozoa on each slide for each sample. The capacitation status was further categorized into four groups: live non-capacitated (F; characterized by green fluorescence evenly distributed over the sperm head), live capacitated (B; with green fluorescence over the acrosome region and a darker post-acrosome region), acrosome-reacted (AR; with no fluorescence over the head), and dead (D; with nuclei showing blue fluorescence within the sperm head) (Kwon et al., 2018).

### 2.6 RNA extraction, cDNA synthesis, and reverse transcription followed by quantitative polymerase chain reaction (RT-qPCR)

RNA extraction, cDNA synthesis, and RT-qPCR were performed according to established procedures (Pang et al., 2020). First, all samples were washed with PBS, centrifuged at 10,000 × g for 10 min, and stored at −80°C prior to RNA extraction. The sperm concentration for each sample was determined and adjusted to 50 × 10^6^ cells/mL with fresh PBS. These samples were then centrifuged at 13,000 × g for 10 min at 4°C to remove the supernatant. Sperm pellets were lysed using lysis buffer (PureLink™ RNA Mini Kit; Invitrogen, Carlsbad, CA, United States) supplemented with 40 μL/mL β-mercaptoethanol (Sigma-Aldrich) and homogenized using 20 G needles. After vigorous mixing of the homogenized solution for 2 min, 500 μL TRIzol reagent (Invitrogen) was added. After 5 min at room temperature, 200 μL of chloroform (Sigma-Aldrich) was added and the solution was thoroughly mixed by inversion for 20 s. After an additional 5 min of incubation at room temperature, the sample was centrifuged at 12,000 × g for 25 min at 4°C. Subsequently, 500 μL of the upper phase containing RNA was carefully transferred to a new 1 mL tube to which an equivalent volume of 100% pure ethanol was added. This mixture was pipetted and processed according to the manufacturer’s instructions. RNA was eluted in 20 μL nuclease-free water. RNA concentrations and 260/280 ratios were quantified using an Epoch microplate spectrophotometer (BioTek, Winooski, VT, United States).

The PrimeScript 1st Strand cDNA Synthesis Kit (Takara Bio, Inc., Shiga, Japan) was used for cDNA synthesis according to the manufacturer’s guidelines. RT-qPCR was performed with gene-specific primers, using glyceraldehyde-3-phosphate dehydrogenase as a reference gene for normalization (Zeng et al., 2014). RT-qPCR data were analyzed using the delta-delta Cq method. The standardized annealing temperature for the designed primers was 60°C. Standard curve analysis was performed for each gene to evaluate PCR efficiency. Melt curve analysis was performed to confirm the presence of a single amplification product, and gel electrophoresis was used to determine the size of the PCR products (Pang et al., 2020).

### 2.7 Statistical analysis

All data were analyzed using SPSS v28 (SPSS Inc., Chicago, IL, United States), and the normality of all parameters was confirmed using the Shapiro-Wilk test. For groups that showed a normal distribution (*p* ≥ 0.05), correlations were determined using Pearson’s correlation coefficients. In cases where normality tests were not passed (*p* < 0.05), Spearman’s correlation coefficient was used (Kwon et al., 2017). Receiver operating characteristic (ROC) curves were used to assess the prognostic potential of all parameters for predicting male fertility and sperm motility, and optimal cut-off values were determined based on the ROC analysis results that yielded the highest sensitivity and specificity values (Swets, 1988). All numerical data are presented as mean ± standard error of the mean, and statistical significance was considered when the *p*-value was less than 0.05.

## 3 Results

### 3.1 Fertility assessment of boar spermatozoa

**FIGURE 1:**
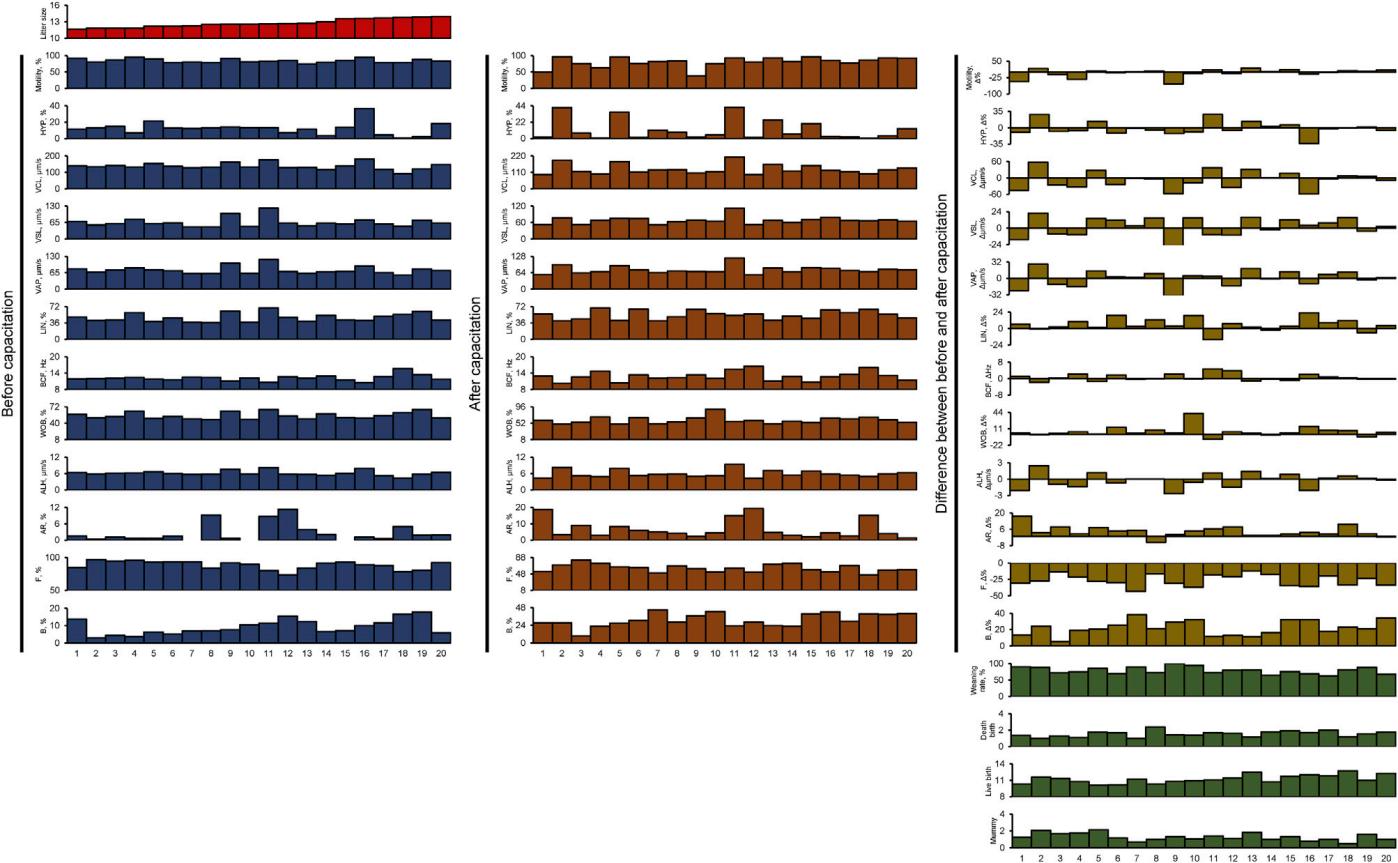
All sperm functional and fertility parameters. HYP, hyperactivated motility; VCL, curvilinear velocity; VSL, straight-line velocity; VAP, average path velocity; BCF, beat cross frequency; LIN, linearity; WOB, wobble; ALH, amplitude of lateral head displacement; AR, acrosome-reacted spermatozoa; F, non-capacitated spermatozoa; B, capacitated spermatozoa; Litter size, total number of pups/total breeding; Weaning rate, percentage of surviving piglets at weaning age (3 weeks); Death birth, number of dead piglets during birth/total breeding; Live birth, total number of living pups/total breeding; Mummy, number of mummified fetuses/total breeding.

We selected 20 boars with well-established AI results, each of which had bred at least 16 sows (mean 22.9 ± 1.3; range between 16 and 38). The litter size of these 20 boars ranged from 11.7 to 14. The fertility of boar spermatozoa was evaluated based on the AI results. The boars were numbered in ascending order of total litter size. In addition, we measured parameters such as weaning rate (%), death births, live births, and mummy births (Figure 1; Supplementary Table S4).

### 3.2 Measurement of the functional parameters of boar spermatozoa

All spermatozoa obtained from the 20 boars had adequate motility, meeting the suitability for use in AI centers (ranging from 74.3% to 95.2%; Figure 1; Supplementary Table S1). In all samples, there was an average increase of 22.1% ± 2.0% in the B pattern, indicating successful *in vitro* capacitation induction (Figure 1; Supplementary Tables S1, S2, S3). Interestingly, although the initial sperm motility was high, the response to *in vitro* capacitation varied between individuals (Figure 1; Supplementary Tables S1, S2, S3).

### 3.3 Sperm functional parameters have not always correlated each other

**FIGURE 2:**
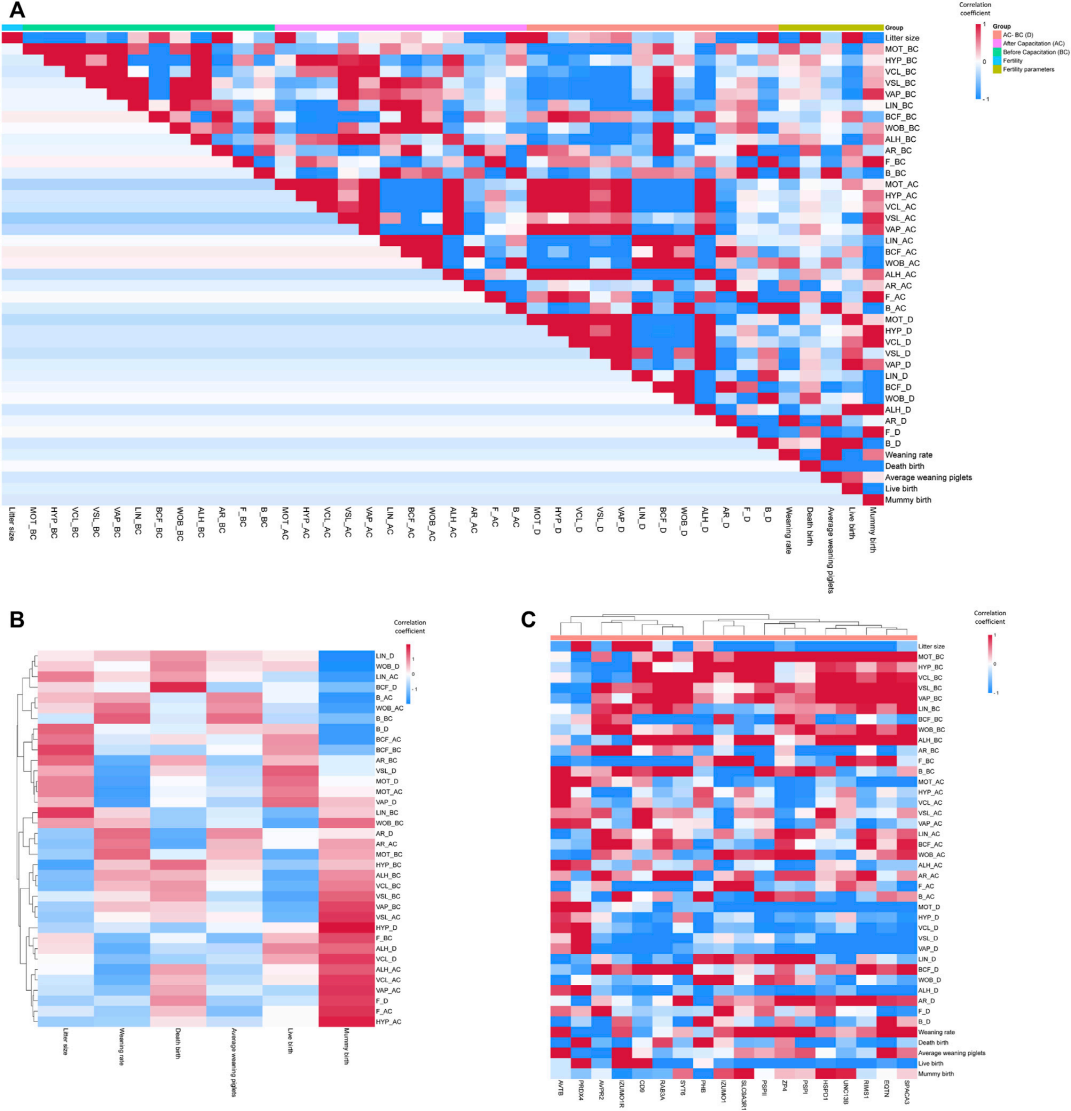
Correlation coefficient heatmap. **(A)** Correlation coefficient heatmap of all sperm functional and fertility parameters. **(B)** Correlation coefficient heatmap of sperm functional *versus* fertility parameters. **(C)** Correlation coefficient heatmap of sperm mRNA expression *versus* functional and fertility parameters. HYP, hyperactivated motility; VCL, curvilinear velocity; VSL, straight-line velocity; VAP, average path velocity; BCF, beat cross frequency; LIN, linearity; WOB, wobble; ALH, amplitude of lateral head displacement; AR, acrosome-reacted spermatozoa; F, non-capacitated spermatozoa; B, capacitated spermatozoa; Litter size, total number of pups/total breeding; Weaning rate, percentage of surviving piglets at weaning age (3 weeks); Death birth, number of dead piglets during birth/total breeding; Average weaning piglet, total number of surviving piglets at weaning age/total breeding; Live birth, total number of living pups/total breeding; Mummy birth, number of mummified fetuses/total breeding.

We evaluated the correlation between all the sperm functional parameters we measured (Figure 2A; Supplementary Table S5). There was a correlation between sperm motility and sperm kinematics (Figure 2A; Supplementary Table S5). In addition, there was a correlation between sperm capacitation status before and after *in vitro* capacitation (Figure 2A; Supplementary Table S5). However, parameters related to sperm motility and capacitation status did not show any correlation with each other (Figure 2A; Supplementary Table S5).

### 3.4 Sperm fertility parameters are correlated with sperm capacitation status after *in vitro* capacitation

The sperm fertility parameters did not show significant correlations with all sperm motility-related parameters (Figure 2A; Supplementary Table S5). In addition, the sperm capacitation status prior to *in vitro* capacitation did not show significant correlations with the sperm fertility parameters (Figure 2A; Supplementary Table S5). Notably, the weaning rate and mummy births correlated with certain parameters of the sperm capacitation status after *in vitro* capacitation. In particular, mummy births showed a significant correlation with the B pattern after *in vitro* capacitation (Figure 2A; Supplementary Table S5). Using hierarchical clustering to analyze sperm functional parameters in a heat map, we observed that most of the correlation coefficients of the motion kinematic parameters related to sperm velocity *versus* mummy births tended to cluster together (Figure 2B; Supplementary Table S5).

### 3.5 Sperm RNA markers are related to sperm function and male fertility

Following the measurement of sperm functional and fertility parameters, we examined the correlations between the expression of sperm RNA markers and each parameter (Figure 2C; Supplementary Table S5). The mRNA expression of HSPD1, IZUMO1, PRDX4, PSP-I, and SLC9A3R1 correlated significantly with litter size (r = −0.44, −0.51, 0.58, 0.52, and −0.71, respectively; Figure 2C; Supplementary Table S5). Sperm motility before *in vitro* capacitation correlated with the mRNA expression of HSPD1, EQTN, UNC13B, PSP-I, and PSP-II (r = 0.67, 0.49, 0.53, 0.50, and 0.52, respectively; Figure 2C; Supplementary Table S5). Although the mRNA expressions of PHB, SPACA3, CD9, and RAB3A did not correlate with sperm motility, they showed correlations with several kinematic parameters prior to *in vitro* capacitation (Figure 2C; Supplementary Table S5).

Surprisingly, the genes that correlated with sperm motility before and after *in vitro* capacitation were different. The mRNA expression of ACTB, ZP4, IZUMO1, and PRDX4 correlated with sperm motility after *in vitro* capacitation (r = 0.57, −0.45, −0.58, and 0.57, respectively; Figure 2C; Supplementary Table S5). The difference in sperm motility between the before and after *in vitro* capacitation values correlated with the mRNA expression of ACTB, ZP4, RIMS1, IZUMO1, and PRDX4 (r = 0.47, −0.50, −0.47, −0.50, and 0.50, respectively; Figure 2C; Supplementary Table S5).

The B pattern of the capacitation status before *in vitro* capacitation was correlated with the mRNA expression of ACTB, IZUMO1, and PSP-II (r = 0.67, −0.50, and 0.59, respectively; Figure 2C; Supplementary Table S5). The mRNA expression of PSP-II was also negatively correlated with the AR pattern before *in vitro* capacitation (r = −0.63; Figure 2C; Supplementary Table S5). The AR pattern after *in vitro* capacitation correlated with the mRNA expression of PHB and SYT6 (*r* = −0.55 and 0.56, respectively; Figure 2C; Supplementary Table S5). The difference in the AR pattern between the before and after *in vitro* capacitation condition correlated with the mRNA expression of SYT6 and PSP-I (*r* = 0.51 and 0.44, respectively; Figure 2C; Supplementary Table S5).

In addition, the weaning rate showed a correlation with PRDX4 mRNA expression (*r* = −0.50, Figure 2C; Supplementary Table S5). We performed hierarchical clustering in a heat map to visualize the correlation coefficients between the sperm functional parameters and sperm RNA expressions (Figure 2C; Supplementary Table S5). Notably, genes with a negative correlation coefficient with litter size tended to have a positive correlation coefficient with most sperm motility-related parameters (Figure 2C; Supplementary Table S5). In addition, genes with a negative correlation coefficient of the difference in motility-related parameters between the before and after capacitation values also showed a negative correlation coefficient (Figure 2C; Supplementary Table S5).

### 3.6 Predictive power of sperm RNA markers for the prognosis of male fertility and sperm motility

**FIGURE 3:**
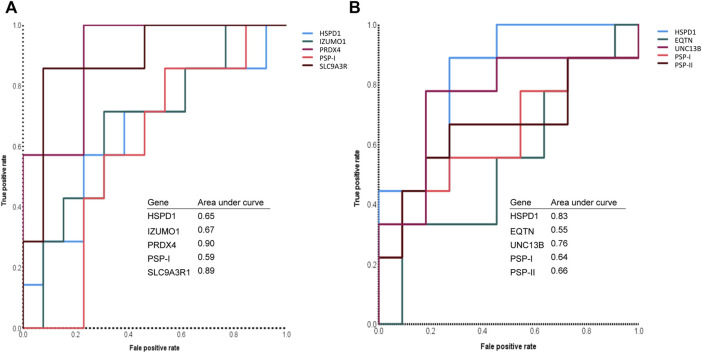
Assessment of sperm mRNA expression to predict male fertility and sperm motility. **(A)** The receiver operating characteristic curve of each sperm RNA marker that can predict the litter size of boars. **(B)** The receiver operating characteristic curve of each sperm RNA marker that can predict the motility of boar spermatozoa.

Finally, we evaluated the predictive power of the sperm RNA markers that showed correlations with male fertility and sperm motility. Using ROC curves, we determined the predictive values of each marker based on cut-off values for male fertility and sperm motility. These cut-offs were calculated as the average of male fertility and sperm motility across all samples. Notably, PRDX4 and SLC9A3R1 showed the highest sensitivity, both at 85.7%, indicating their robust predictive potential (Figure 3; Table 1). In particular, SLC9A3R1 showed an impressive overall accuracy of 85% for predicting male fertility, outperforming the other genes (Figure 3; Table 1).

**TABLE 1:** Male fertility prediction value of sperm RNA markers. Sensitivity is the percentage of pigs showing true-positive results when tested with mRNA expression. Specificity is the percentage of pigs showing true-negative results. The positive predictive value (PPV) is the percentage of pigs that tested as positive and simultaneously showed true-positive litter size. The negative predictive value (NPV) is the percentage of pigs that tested as negative and simultaneously showed true-negative litter size. OA overall accuracy.

Gene	Sensitivity, %	Specificity, %	NPV, %	PPV, %	OA, %
HSPD1	57.1	61.5	72.7	44.4	60.0
IZUMO1	71.4	69.2	81.8	55.6	70.0
PRDX4	85.7	69.2	90.0	60.0	75.0
PSP-I	57.1	61.5	72.7	44.4	60.0
SLC9A3R1	85.7	84.6	91.7	75.0	85.0

It is noteworthy that the overall accuracy of the tested sperm RNA markers for predicting sperm motility was slightly lower than for predicting male fertility (Tables 1, 2). Among these markers, UNC13B emerged as the most accurate for predicting sperm motility with an accuracy rate of 80%. Furthermore, UNC13B had the highest sensitivity of 77.8% (Figure 3; Table 2).

**TABLE 2:** Sperm motility prediction value of sperm RNA markers. Sensitivity is the percentage of pigs showing true-positive results when tested with mRNA expression. Specificity is the percentage of pigs showing true-negative results. The positive predictive value (PPV) is the percentage of pigs that tested as positive and simultaneously showed true-positive litter size. The negative predictive value (NPV) is the percentage of pigs that tested as negative and simultaneously showed true-negative litter size. OA overall accuracy.

Gene	Sensitivity, %	Specificity, %	NPV, %	PPV, %	OA, %
HSPD1	66.7	72.7	72.7	66.7	70.0
EQTN	55.6	54.5	60.0	50.0	55.0
UNC13B	77.8	81.8	81.8	77.8	80.0
PSP-I	55.6	54.5	60.0	50.0	55.0
PSP-II	66.7	63.6	70.0	60.0	65.0

yHSPD1 and PSP-I showed correlations with both male fertility and sperm motility (Supplementary Table S5). Interestingly, both genes showed the same overall accuracy of 60% for predicting male fertility (Figure 3; Table 1). However, when predicting sperm motility, HSPD1 showed an overall accuracy that was 15% higher than that of PSP-I (Figure 3; Table 2).

## 4 Discussion

Assessment of sperm quality is critical to the diagnosis and prediction of male infertility. While the current biomedical evaluation, including the WHO criteria, focuses primarily on sperm motility, it falls short of capturing motility changes within the female reproductive tract. This calls for a more comprehensive method to accurately diagnose male infertility. While numerous techniques have been developed to assess sperm motility, it is clear that sperm motility alone is insufficient to explain the totality of male infertility and remains an ongoing challenge. Instead of relying solely on sperm functional parameters, researchers are turning to various markers, with a particular focus on transcriptomic markers. These markers are promising due to advanced high-throughput analysis techniques and accessible validation tools. Recent studies have explored the predictive potential of sperm RNA for fertility, including specific genes in porcine sperm.

In this study, we conducted the comprehensive assessment of sperm RNA as a potential factor to diagnose or prognose sperm function from motility to beyond fertilization. As a sperm functional parameter, we measured sperm motility and motion kinematics using the Computer-Assisted Sperm Analysis system before and after *in vitro* capacitation. Using boar spermatozoa, we acquired male fertility data from the litter size after AI. Moreover, the weaning rate, death births, live births, and mummy births were checked to reflect the biological aspect after the beyond fertilization point.

Correlation analysis revealed several interesting relationships between the sperm functional parameters. The observed correlation between sperm motility and sperm kinematics underscores the interdependence of these parameters in predicting sperm motility-related function. Furthermore, the correlation between sperm capacitation status before and after *in vitro* capacitation highlights the importance of assessing the sperm capacitation ability as a key functional parameter. Surprisingly, none of the functional parameters were correlated with litter size. This supports the reason the practical application of sperm motility-related parameters to predict male fertility is controversial (Wang and Swerdloff, 2014; Patel et al., 2018; Lamb and Marinaro, 2023). Instead of litter size, we found a correlation between specific parameters of sperm capacitation status after *in vitro* capacitation and fertility parameters such as the weaning rate and mummy births. This finding emphasizes a link between sperm capacitation and successful fertilization (Austin, 1951; Austin, 1952; Rahman et al., 2017). Furthermore, given the associations between weaning rates, mummy births, and sperm capacitation, it is reasonable to speculate that proper sperm capacitation may influence developmental processes beyond fertilization.

All the genes which underwent sperm RNA marker assessment in this study are already known to be related to pig fertility. We checked the mRNA expression of the corresponding genes in the spermatozoa, which have well-organized male fertility data. Using these gene expression levels, we assessed whether they could diagnose and prognose all sperm functional and fertility parameters. The investigation into the relationships between sperm RNA markers and functional/fertility parameters revealed intriguing associations. The correlation of certain sperm RNA markers with litter size, sperm motility before *in vitro* capacitation, and kinematic parameters underscores the potential role of these genes in sperm function and fertility. Particularly, IZUMO1, which is a protein known to have a role during mammalian sperm-egg fusion (Hernandez-Falco et al., 2022), was linked to motility, capacitation, and fertilization in this study. These data provide new insight that IZUMO1 may have a functional role not only in sperm-egg fusion in mammalian spermatozoa, but also the reliability of other data that genes proven to correlate with sperm functional parameters in the current study are important targets for studying male fertility. Similar to IZUMO1, CD9 is a critical tetraspanin protein known for its involvement in sperm-egg fusion (Kaji et al., 2000; Li et al., 2004). However, in our study, CD9 did not show a significant correlation with fertility parameters. Instead, we observed correlations between CD9 and the parameters VCL, BCF, and ALH, which are associated with hyperactivated motility. It is worth noting that our CASA system determines hyperactivated motility based on the straightness of sperm movement rather than BCF and ALH. Consequently, the correlation coefficient between CD9 expression and hyperactivated motility did not reach statistical significance. Nevertheless, our results provide insight into the potential mechanisms of CD9 in relation to hyperactivated motility and highlight possible species-specific differences in its function.

In this study, we rigorously evaluated the predictive value of each sperm RNA marker through comprehensive statistical analyses using results from AI with corresponding pig spermatozoa. In particular, our investigation identified two mRNA expressions, HSPD1 and PSP-I, as candidates with the potential to serve as markers of both male fertility and sperm motility. It is worth noting that these genes are already recognized as markers of sperm function at the protein level (Mitchell et al., 2007; Novak et al., 2010; De Lazari et al., 2019; Albrizio et al., 2020). HSPD1 codes for mitochondrial molecular chaperone protein which is essential for protein folding. Recent structural analysis of HSPD1 using cryo-EM aided its importance in protein folding (Klebl et al., 2021). Because spermatozoa is known to have mitochondrial translation (Gur and Breitbart, 2006), HSPD1 will also affect correct protein folding of these proteins and consequently affect sperm functions. The PSP-I is spermadhesin mainly found in seminal plasma of boar and has possible role in sperm-egg interaction (Caballero et al., 2005). As the main expression site of PSP-I is seminal plasma, the mRNA in sperm might originated from male reproductive tract during sperm maturation. Kang et al. had investigated predictive power of PSP-I mRNA in boar spermatozoa (Kang et al., 2019). Although the predictive value was different, study from Kang et al. and present study showed same negative correlation with litter size. Although the predictive value of both genes was comparatively low, their importance in controlling processes from sperm motility to fertilization is undeniable.

Among sperm RNA markers, SLC9A3R1 emerged as the best marker of male fertility, while UNC13B took the lead in predicting sperm motility. SLC9A3R1 encodes a modulator of a Na+/H+ exchange membrane protein (Kreimann et al., 2015). In mouse spermatozoa, SLC9A3R1 plays a role in ion exchange associated with capacitation (Chavez et al., 2012). However, our present study suggests that SLC9A3R1 may have a broader role beyond capacitation. The UNC13B protein, which is primarily found in neurons and is associated with vesicle maturation through its diacylglycerol (DAG) domain (Wang et al., 2019), is closely related to the acrosome reaction in mammalian spermatozoa, a crucial process for normal fertilization. In our current study, we found a correlation between UNC13B mRNA expression and sperm motility. These findings shed light on the novel roles these genes play in mammalian spermatozoa and their contributions to male fertility. Importantly, the overall accuracy in predicting either male fertility or sperm motility fell below the 90% threshold, underscoring the need to combine multiple methods for accurate diagnosis of male infertility.

Traditional semen analysis in various mammalian species primarily involves the assessment of sperm morphology and motility. When sperm morphology was used to predict male fertility in bulls and boars, the AUC in the ROC analysis ranged from 0.54 to 0.60 (Barquero et al., 2021; Llavanera et al., 2021). In human and bovine, the AUC for sperm motility in assessing male fertility was 0.62 and 0.68, respectively (Jeong et al., 2021; Llavanera et al., 2021). Our study demonstrated AUC values ranging from 0.65 to 0.90 for individual genes in predicting male fertility. These values are comparatively higher than those achieved by conventional semen/sperm analysis parameters. However, it is important to note that both functional parameters and sperm RNA markers have room for further refinement. We propose that the combination of both methods can significantly improve the accuracy of male fertility prediction.

While the importance of sperm RNA in fertilization has been recognized, its function has remained controversial due to its complex nature. The RNA content of sperm consists of both residual RNA from the spermatogenesis and sperm-borne RNA (Chen et al., 2016). Considering that sperm are known for their cessation of DNA transcription, sperm mRNA may serve as a reflection of the final transcriptional phase during spermatogenesis. The limitation of the current study is that we did not aim to provide a comprehensive overview of the fundamental role of sperm mRNA. Because we focused on the mRNA expression of specific genes in spermatozoa, we propose that the potential roles of these genes may contribute to fertilization in two ways: 1) Involvement in biochemical changes associated with the respective genes (Gur and Breitbart, 2006) and 2) Involvement in post-fertilization processes after delivery to the oocyte (Bayer et al., 2009). The correlations between sperm mRNA expression and function demonstrated in this study provide valuable insights into understanding biomolecular changes in sperm, which in turn can significantly advance male fertility research.

## 5 Conclusion

According to the present study, sperm RNA serves as a valuable marker for assessing sperm function, which includes aspects from motility to fertility. It is evident that the mRNA of specific genes in sperm can be used for both the diagnosis and prognosis of sperm function. Any RNA detection method can be widely used to detect the markers identified here, potentially paving the way for the development of diagnostic methods for male infertility. Furthermore, in the context of sperm RNA research, we propose that investigating the role of sperm RNA may provide new insights into our understanding of male infertility.

## Data Availability

The original contributions presented in the study are included in the article/Supplementary Material, further inquiries can be directed to the corresponding author.
