# Supplementary material for: Development of a biomolecular approach to identify sperm functions and fertility using sperm RNAs
Source: Front Cell Dev Biol. 2023 Dec 5;11:1308167. doi: 10.3389/fcell.2023.1308167 (PMC10728490; doi:10.3389/fcell.2023.1308167)
Supplement: Supplementary file 1 [file Presentation1.zip › Suppl. Table 1- 4.DOCX]

Supplementary Material

## Supplementary Tables

**Supplementary Table S1.** All functional parameters of boar spermatozoa before in vitro capacitation induction. HYP, hyperactivated motility; VCL, curvilinear velocity; VSL, straight-line velocity; VAP, average path velocity; BCF, beat cross frequency; LIN, linearity; WOB, wobble; ALH, amplitude of lateral head displacement; AR, acrosome-reacted spermatozoa; F, non-capacitated spermatozoa; B, capacitated spermatozoa.

|  | Sperm motility and motion kinetics | | | | | | | | |  | Capacitation status | | |
| --- | --- | --- | --- | --- | --- | --- | --- | --- | --- | --- | --- | --- | --- |
| Sample number | Motility, % | HYP, % | VCL, μm/s | VSL, μm/s | VAP, μm/s | LIN, % | BCF, Hz | WOB, % | ALH, μm/s |  | AR, % | F, % | B, % |
| 1 | 92.1 | 11.5 | 140.5 | 69.0 | 80.7 | 49.1 | 11.8 | 57.5 | 6.4 |  | 1.5 | 84.8 | 13.7 |
| 2 | 80.2 | 13.1 | 133.4 | 55.3 | 67.6 | 41.4 | 11.9 | 50.7 | 5.8 |  | 0.3 | 96.8 | 2.9 |
| 3 | 86.6 | 14.8 | 142.3 | 60.7 | 75.6 | 42.6 | 12.2 | 53.1 | 6.2 |  | 1.1 | 94.7 | 4.3 |
| 4 | 95.2 | 6.9 | 132.7 | 77.8 | 84.4 | 58.7 | 12.4 | 63.7 | 6.2 |  | 0.7 | 95.6 | 3.7 |
| 5 | 90.2 | 21.5 | 154.8 | 61.1 | 76.5 | 39.5 | 11.8 | 49.4 | 6.7 |  | 0.7 | 93.1 | 6.3 |
| 6 | 79.0 | 13.0 | 136.7 | 63.2 | 72.8 | 46.9 | 11.6 | 53.2 | 6.0 |  | 1.5 | 93.3 | 5.2 |
| 7 | 80.1 | 12.2 | 128.2 | 47.9 | 62.2 | 37.3 | 12.5 | 48.5 | 5.7 |  | 0.0 | 93.1 | 6.9 |
| 8 | 79.0 | 13.2 | 131.6 | 48.4 | 62.2 | 36.8 | 12.4 | 47.3 | 5.8 |  | 9.2 | 83.8 | 7.0 |
| 9 | 91.8 | 14.1 | 162.3 | 101.1 | 103.4 | 62.3 | 11.1 | 63.7 | 7.6 |  | 0.7 | 91.7 | 7.6 |
| 10 | 80.5 | 13.5 | 132.7 | 49.9 | 63.5 | 37.6 | 12.3 | 47.8 | 5.8 |  | 0.0 | 89.6 | 10.4 |
| 11 | 82.8 | 13.5 | 175.9 | 121.6 | 117.5 | 68.9 | 10.7 | 66.7 | 8.2 |  | 8.7 | 80.0 | 11.4 |
| 12 | 85.5 | 7.1 | 128.8 | 62.2 | 70.0 | 48.3 | 12.6 | 54.3 | 5.8 |  | 11.3 | 73.3 | 15.4 |
| 13 | 74.3 | 11.3 | 130.8 | 52.1 | 63.5 | 39.8 | 12.2 | 48.5 | 5.7 |  | 3.9 | 83.8 | 12.3 |
| 14 | 79.5 | 3.5 | 117.8 | 62.2 | 69.0 | 52.8 | 12.9 | 58.6 | 5.4 |  | 2.1 | 91.4 | 6.5 |
| 15 | 85.6 | 13.9 | 138.8 | 59.3 | 70.9 | 42.7 | 11.5 | 51.1 | 6.1 |  | 0.0 | 92.9 | 7.1 |
| 16 | 94.9 | 36.6 | 181.9 | 75.3 | 91.2 | 41.4 | 10.5 | 50.2 | 7.9 |  | 1.1 | 88.9 | 10.0 |
| 17 | 79.3 | 4.4 | 118.4 | 59.6 | 65.3 | 50.3 | 12.7 | 55.2 | 5.2 |  | 0.6 | 87.9 | 11.6 |
| 18 | 79.3 | 0.9 | 92.4 | 50.4 | 56.1 | 54.6 | 15.6 | 60.7 | 4.4 |  | 4.9 | 78.4 | 16.6 |
| 19 | 88.5 | 2.3 | 120.4 | 74.0 | 80.3 | 61.5 | 13.4 | 66.8 | 5.8 |  | 1.8 | 80.3 | 17.9 |
| 20 | 83.4 | 18.4 | 147.2 | 62.3 | 74.1 | 42.3 | 11.7 | 50.3 | 6.4 |  | 1.9 | 92.2 | 5.9 |

**Supplementary Table S2.** All functional parameters of boar spermatozoa after in vitro capacitation induction. HYP, hyperactivated motility; VCL, curvilinear velocity; VSL, straight-line velocity; VAP, average path velocity; BCF, beat cross frequency; LIN, linearity; WOB, wobble; ALH, amplitude of lateral head displacement; AR, acrosome-reacted spermatozoa; F, non-capacitated spermatozoa; B, capacitated spermatozoa.

|  | Sperm motility and motion kinetics | | | | | | | | |  | Capacitation status | | |
| --- | --- | --- | --- | --- | --- | --- | --- | --- | --- | --- | --- | --- | --- |
| Sample number | Motility, % | HYP, % | VCL, μm/s | VSL, μm/s | VAP, μm/s | LIN, % | BCF, Hz | WOB, % | ALH, μm/s |  | AR, % | F, % | B, % |
| 1 | 49.5 | 1.7 | 94.7 | 52.5 | 56.6 | 55.5 | 13.0 | 59.9 | 4.3 |  | 18.7 | 54.2 | 27.1 |
| 2 | 96.7 | 41.4 | 190.5 | 77.3 | 95.4 | 40.6 | 10.2 | 50.1 | 8.3 |  | 3.3 | 69.5 | 27.2 |
| 3 | 75.3 | 7.3 | 116.4 | 52.4 | 63.9 | 45.0 | 12.5 | 54.9 | 5.3 |  | 9.0 | 81.5 | 9.5 |
| 4 | 62.9 | 0.8 | 99.3 | 68.2 | 68.1 | 68.8 | 14.7 | 68.8 | 4.8 |  | 2.9 | 74.3 | 22.8 |
| 5 | 96.1 | 35.3 | 182.1 | 75.1 | 91.1 | 41.3 | 10.4 | 50.1 | 7.9 |  | 8.2 | 65.1 | 26.7 |
| 6 | 75.7 | 1.6 | 112.8 | 74.8 | 75.7 | 66.3 | 13.3 | 67.2 | 5.3 |  | 6.0 | 63.4 | 30.6 |
| 7 | 81.9 | 11.2 | 127.1 | 51.4 | 64.5 | 40.5 | 12.2 | 50.7 | 5.7 |  | 5.1 | 50.0 | 44.9 |
| 8 | 83.9 | 8.5 | 127.7 | 63.2 | 71.5 | 49.5 | 12.4 | 56.1 | 5.8 |  | 4.0 | 67.6 | 28.3 |
| 9 | 38.0 | 1.9 | 104.6 | 68.7 | 69.2 | 65.9 | 13.3 | 66.4 | 5.0 |  | 2.3 | 60.8 | 36.9 |
| 10 | 75.1 | 4.8 | 114.6 | 64.9 | 68.5 | 56.6 | 12.1 | 89.7 | 5.2 |  | 4.4 | 52.8 | 42.8 |
| 11 | 92.5 | 41.9 | 213.1 | 112.0 | 121.5 | 52.6 | 15.4 | 57.0 | 9.3 |  | 15.0 | 61.7 | 23.3 |
| 12 | 80.2 | 1.6 | 93.9 | 52.1 | 55.7 | 55.5 | 16.5 | 59.3 | 4.3 |  | 19.3 | 52.4 | 28.3 |
| 13 | 92.7 | 24.9 | 162.1 | 67.9 | 82.4 | 41.9 | 11.1 | 50.9 | 7.1 |  | 4.7 | 71.7 | 23.6 |
| 14 | 81.8 | 6.1 | 118.4 | 60.2 | 67.8 | 50.8 | 12.7 | 57.3 | 5.5 |  | 2.9 | 74.2 | 22.9 |
| 15 | 96.5 | 20.1 | 155.6 | 71.5 | 83.7 | 45.9 | 10.8 | 53.8 | 6.9 |  | 2.1 | 58.4 | 39.5 |
| 16 | 85.7 | 2.8 | 123.2 | 79.3 | 81.2 | 64.4 | 12.6 | 65.9 | 5.8 |  | 4.4 | 53.4 | 42.3 |
| 17 | 77.4 | 2.2 | 114.4 | 67.1 | 72.7 | 58.6 | 13.5 | 63.6 | 5.4 |  | 2.5 | 68.2 | 29.2 |
| 18 | 86.1 | 0.6 | 99.9 | 65.9 | 68.3 | 66.0 | 16.1 | 68.4 | 4.9 |  | 15.2 | 45.1 | 39.6 |
| 19 | 92.9 | 3.7 | 126.3 | 69.7 | 77.9 | 55.2 | 13.1 | 61.8 | 5.9 |  | 4.0 | 57.0 | 38.9 |
| 20 | 92.2 | 13.2 | 138.8 | 64.8 | 75.5 | 46.7 | 11.5 | 54.4 | 6.3 |  | 1.4 | 58.5 | 40.1 |

**Supplementary Table S3.** Difference in functional parameters of boar spermatozoa before and after in vitro capacitation induction. HYP, hyperactivated motility; VCL, curvilinear velocity; VSL, straight-line velocity; VAP, average path velocity; BCF, beat cross frequency; LIN, linearity; WOB, wobble; ALH, amplitude of lateral head displacement; AR, acrosome-reacted spermatozoa; F, non-capacitated spermatozoa; B, capacitated spermatozoa.

|  | Sperm motility and motion kinetics | | | | | | | | |  | Capacitation status | | |
| --- | --- | --- | --- | --- | --- | --- | --- | --- | --- | --- | --- | --- | --- |
| Sample number | Motility, % | HYP, % | VCL, μm/s | VSL, μm/s | VAP, μm/s | LIN, % | BCF, Hz | WOB, % | ALH, μm/s |  | AR, % | F, % | B, % |
| 1 | -42.7 | -9.7 | -45.8 | -16.6 | -24.1 | 6.3 | 1.1 | 2.4 | -2.1 |  | 17.2 | -30.6 | 13.4 |
| 2 | 16.5 | 28.3 | 57.1 | 21.9 | 27.7 | -0.9 | -1.7 | -0.6 | 2.5 |  | 3.0 | -27.3 | 24.3 |
| 3 | -11.3 | -7.6 | -26.0 | -8.3 | -11.7 | 2.4 | 0.4 | 1.8 | -0.9 |  | 7.9 | -13.2 | 5.2 |
| 4 | -32.3 | -6.1 | -33.4 | -9.6 | -16.3 | 10.1 | 2.3 | 5.1 | -1.4 |  | 2.2 | -21.3 | 19.1 |
| 5 | 5.8 | 13.8 | 27.2 | 14.0 | 14.6 | 1.8 | -1.4 | 0.7 | 1.2 |  | 7.5 | -28.0 | 20.5 |
| 6 | -3.3 | -11.3 | -23.9 | 11.6 | 2.9 | 19.4 | 1.7 | 13.9 | -0.7 |  | 4.5 | -29.9 | 25.4 |
| 7 | 1.8 | -1.0 | -1.1 | 3.6 | 2.3 | 3.2 | -0.3 | 2.2 | 0.0 |  | 5.1 | -43.1 | 38.1 |
| 8 | 4.9 | -4.7 | -4.0 | 14.9 | 9.4 | 12.8 | 0.0 | 8.8 | 0.0 |  | -5.1 | -16.2 | 21.3 |
| 9 | -53.8 | -12.2 | -57.7 | -32.4 | -34.1 | 3.6 | 2.3 | 2.7 | -2.7 |  | 1.6 | -31.0 | 29.3 |
| 10 | -5.4 | -8.7 | -18.0 | 15.0 | 5.0 | 19.0 | -0.2 | 41.9 | -0.6 |  | 4.4 | -36.8 | 32.4 |
| 11 | 9.7 | 28.4 | 37.2 | -9.6 | 4.0 | -16.3 | 4.7 | -9.7 | 1.1 |  | 6.3 | -18.2 | 11.9 |
| 12 | -5.3 | -5.5 | -34.9 | -10.1 | -14.3 | 7.2 | 3.8 | 5.0 | -1.5 |  | 8.0 | -20.9 | 12.9 |
| 13 | 18.4 | 13.6 | 31.3 | 15.8 | 18.9 | 2.1 | -1.1 | 2.3 | 1.4 |  | 0.8 | -12.1 | 11.3 |
| 14 | 2.3 | 2.6 | 0.6 | -2.0 | -1.2 | -2.0 | -0.2 | -1.4 | 0.1 |  | 0.8 | -17.3 | 16.4 |
| 15 | 11.0 | 6.2 | 16.9 | 12.2 | 12.8 | 3.3 | -0.7 | 2.7 | 0.9 |  | 2.1 | -34.4 | 32.4 |
| 16 | -9.1 | -33.9 | -58.7 | 4.0 | -10.1 | 23.0 | 2.1 | 15.8 | -2.0 |  | 3.3 | -35.6 | 32.3 |
| 17 | -1.9 | -2.3 | -4.0 | 7.4 | 7.4 | 8.3 | 0.8 | 8.4 | 0.2 |  | 2.0 | -19.6 | 17.7 |
| 18 | 6.8 | -0.3 | 7.5 | 15.5 | 12.2 | 11.4 | 0.4 | 7.7 | 0.6 |  | 10.3 | -33.3 | 23.0 |
| 19 | 4.4 | 1.4 | 5.9 | -4.3 | -2.4 | -6.2 | -0.3 | -5.0 | 0.1 |  | 2.2 | -23.2 | 21.0 |
| 20 | 8.8 | -5.2 | -8.3 | 2.5 | 1.4 | 4.4 | -0.3 | 4.0 | -0.2 |  | -0.5 | -33.7 | 34.2 |

**Supplementary Table S4.** Fertility parameters of boar spermatozoa.

Litter size, total number of pups/total breeding; Weaning rate, percentage of surviving piglets at weaning age (3 weeks); Death birth, number of dead piglets during birth/total breeding; Average weaning piglet, total number of surviving piglets at weaning age/ total breeding; Live birth, total number of living pups/total breeding; Mummy birth, number of mummified fetuses/total breeding.

| Sample number (the number of semen samples used) | Litter size | Weaning rate, % | Death birth | Average weaning piglet | Live birth | Mummy birth |
| --- | --- | --- | --- | --- | --- | --- |
| 1 (76) | 11.7 | 90.3 | 1.4 | 10.5 | 10.3 | 1.3 |
| 2 (68) | 11.8 | 88.3 | 1.0 | 11.1 | 11.6 | 2.1 |
| 3 (36) | 11.8 | 71.4 | 1.3 | 9.0 | 11.3 | 1.7 |
| 4 (48) | 11.8 | 75.1 | 1.1 | 8.9 | 10.7 | 1.8 |
| 5 (60) | 12.2 | 85.0 | 1.8 | 10.1 | 10.1 | 2.1 |
| 6 (42) | 12.2 | 69.4 | 1.7 | 8.2 | 10.2 | 1.2 |
| 7 (28) | 12.3 | 89.4 | 1.0 | 10.9 | 11.2 | 0.7 |
| 8 (32) | 12.5 | 72.2 | 2.4 | 9.2 | 10.3 | 1.0 |
| 9 (44) | 12.5 | 100.0 | 1.4 | 12.2 | 10.8 | 1.3 |
| 10 (50) | 12.6 | 94.5 | 1.4 | 11.6 | 10.9 | 1.1 |
| 11 (36) | 12.6 | 72.3 | 1.7 | 9.2 | 11.1 | 1.4 |
| 12 (38) | 12.7 | 79.7 | 1.6 | 10.4 | 11.4 | 1.1 |
| 13 (38) | 12.7 | 80.2 | 1.1 | 10.9 | 12.5 | 1.8 |
| 14 (50) | 13.0 | 64.5 | 1.8 | 8.1 | 10.7 | 1.0 |
| 15 (46) | 13.6 | 75.7 | 1.9 | 10.3 | 11.7 | 1.3 |
| 16 (50) | 13.6 | 68.8 | 1.7 | 9.4 | 12.0 | 0.8 |
| 17 (38) | 13.7 | 62.2 | 2.0 | 8.6 | 11.8 | 1.0 |
| 18 (36) | 13.8 | 81.0 | 1.2 | 11.3 | 12.7 | 0.5 |
| 19 (38) | 13.9 | 88.3 | 1.5 | 11.1 | 11.0 | 1.6 |
| 20 (36) | 14.0 | 67.4 | 1.8 | 9.4 | 12.3 | 1.0 |

**Supplementary Table S5.** Correlation coefficients for all correlation analyses. * P < 0.05.

Supplementary Table S5 is provided as a separate .xisx file due to its size.
